# Utilization patterns and prescription characteristics of traditional Chinese medicine among patients with irritable bowel syndrome in Taiwan

**DOI:** 10.3389/fphar.2023.1201240

**Published:** 2023-06-16

**Authors:** Ye Gu, Yu-Tung Lai, Fang-Rong Chang, Chung-Yu Chen

**Affiliations:** ^1^ Graduate Institute of Natural Products, College of Pharmacy, Kaohsiung Medical University, Kaohsiung, Taiwan; ^2^ Master Program in Clinical Pharmacy, School of Pharmacy, Kaohsiung Medical University, Kaohsiung, Taiwan; ^3^ Department of Medical Research, Kaohsiung Medical University Hospital, Kaohsiung, Taiwan; ^4^ Drug Development and Value Creation Research Center, Kaohsiung Medical University Hospital, Kaohsiung, Taiwan; ^5^ Department of Pharmacy, Kaohsiung Medical University Hospital, Kaohsiung, Taiwan

**Keywords:** traditional Chinese medicine, irritable bowel syndrome (IBS), Chinese herbal medicine, utilization patterns, real-world data

## Abstract

**Background:** Few studies have investigated traditional Chinese medicine (TCM) utilization patterns for irritable bowel syndrome (IBS), despite the potential benefits of exploring TCM utilization patterns in optimizing TCM management. This study aimed to evaluate TCM utilization patterns and clinical features for IBS patterns in Taiwan.

**Methods:** This was a population-based cross-sectional study using claim data from the National Health Insurance Research Database between 2012 and 2018. Patients newly diagnosed with IBS and aged over 20 years were included. The TCM utilization patterns and characteristics, including Chinese herbal medicine (CHM) treatment types and prescription patterns, were evaluated.

**Results:** A total of 73,306 patients newly diagnosed with IBS used TCM for IBS at least once. Females used TCM for IBS more than males (female-to-male ratio = 1.89: 1). The age distribution showed a peak at 30–39 years (27.29%), followed by 40–49 years (20.74%) and 20–29 years (20.71%). Patients who received Western medications for IBS had a lower tendency to seek TCM. CHM was the most commonly used TCM modality (98.22%), with Jia-wei-xiao-yao-san being the most commonly prescribed Chinese herbal formula and Bai-zhu being the most frequently prescribed single Chinese herb.

**Conclusion:** This study enhances our understanding of TCM usage patterns for IBS, particularly CHM prescriptions. Further research is needed to investigate commonly used TCM formulas and individual herbs.

## Introduction

Irritable bowel syndrome (IBS) is a prevalent chronic gastrointestinal (GI) disorder affecting around 11.2% of the global population (Lovell and Ford, 2012). In Taiwan, the average annual incidence and prevalence showed a decreasing trend from 2012 to 2018, the use of International Classification of Diseases (ICD) codes rather than rigorous research criteria to identify IBS diagnoses may have led to upcoding or misdiagnosis, which could have affected the reported incidence and prevalence of IBS (Lai et al., 2021). Despite the high prevalence of IBS, its pathophysiology remains incompletely understood (Soares, 2014). The IBS symptoms include chronic or recurring abdominal pain and changes in bowel habits without any structural or biochemical abnormalities or the presence of any organic cause (Masuy et al., 2020). IBS management aims to alleviate symptoms and improve the quality of life, since the condition can negatively impact mental and physical wellbeing (Black and Ford, 2020). Treatment typically involves a combination of psychological support, lifestyle and dietary modifications, physical activity, and pharmaceutical interventions. Non-pharmaceutical treatments are recommended as the first-line approach and can be tailored to the individual’s predominant symptoms such as stress management and dietary modifications for bowel symptoms (Smith, 2006). Pharmaceutical treatment is considered for patients who do not respond to non-pharmaceutical therapies. Beneficial to non-GI symptoms and comorbidities, which can improve health-related quality of life and symptom severity (Gwee et al., 2019; Moayyedi et al., 2019; Masuy et al., 2020; Fukudo et al., 2021; Lacy et al., 2021). Many patients with IBS are unsatisfied with conventional treatment and seek complementary and alternative medicine (CAM) (Li and Li, 2015; Ford et al., 2017), such as traditional Chinese medicine (TCM), which is commonly used in East Asia for IBS treatment (Wu et al., 2021). High-quality studies suggest that TCM formulas, including Tong-xie-yao-fang granule and Shen-ling-bai-zhu-san, effectively manage IBS global symptoms (M Chen et al., 2018; M. Chen et al., 2018; Wang et al., 2020; Xiao et al., 2015), but their use lacks consensus and mainly depends on experiences of TCM practitioners. Thus, this cross-sectional study aimed to investigate TCM utilization patterns and clinical characteristics among patients newly diagnosed with IBS to address the knowledge gap regarding TCM use in IBS treatment in Taiwan.

## Methods

### Data sources

This was a population-based study using data from the National Health Insurance Research Database (NHIRD) in Taiwan from 2011 to 2018. The National Health Insurance (NHI) program offers universal health insurance to nearly all of the local population, and the NHIRD holds complete and excellent data on demographics, clinical visits, hospitalizations, and prescriptions. The diagnoses are based on the International Classification of Diseases, Ninth and 10th Editions and Clinical Modification (ICD-9-CM and ICD-10-CM) codes. The prescriptions, including Chinese herbal medicine (CHM), contain generic names, brand names, and dosages. This study was approved by the Institutional Review Board (IRB) of Kaohsiung Medical University Chung-Ho Memorial Hospital [KMUHIRB-E(II)-20190359]. Individual identification numbers were encrypted with unique and anonymous identifiers to protect privacy; thus, the requirement for consent was waived by the IRB.

### Study population

Patients aged 20 years or above with at least one outpatient visit or hospitalization for IBS (ICD-9-CM code: 564.1 and ICD-10-CM code: K58.0 and K58.9) from 2012 to 2017 were enrolled. Exclusion criteria included patients with missing age and gender information and those with a prior IBS diagnosis before 2012 to ensure that all patients were newly diagnosed with IBS. Patients were then categorized as either TCM or non-TCM users. TCM users were defined as those who received an IBS diagnosis after their initial diagnosis and sought for treatment at TCM clinics.

### Study variables

Data, including demographics, comorbidities, and comedications, were collected to analyze the independent variables that influence the use of TCM for IBS. Patients were divided into six age groups for both genders (20–29, 30–39, 40–49, 50–59, 60–69, and ≥70). Urbanization levels in Taiwan were classified into seven tiers, with 1 being the most urbanized and 7 being the least. Residential areas were categorized as levels 1 and 2 (urban areas), levels 3 and 4 (suburban areas), and levels 5 and above (rural areas). Insurance premiums in New Taiwan Dollars (NT$) are determined based on the monthly income of the insured individual and can be used as an indicator of their economic status. The premiums are categorized into three groups: <NT$18,780, NT$18,780–NT$29,000, and >NT$29,000 in this study. This study investigated the comorbidities and prescribed comedication in IBS patients to better understand their disease status. Comorbidities were defined as having at least two ambulatory or outpatient diagnoses or one inpatient diagnosis before IBS diagnosis and identified using the ICD-9-CM and ICD-10-CM codes (Supplementary Table S1). Comedications were defined as the use of medications of interest for a minimum of 14 days before IBS diagnosis and identified using Anatomical Therapeutic Chemical codes, which included laxatives, antidiarrheals, antispasmodics, antidepressants, and probiotics (Supplementary Table S2). The NHIRD provides detailed information on TCM utilization, including TCM diagnoses, CHM prescription, and related claim data. A list of reimbursed CHM was obtained from the Taiwan National Health Insurance Administration website, including NHI codes and names of Chinese herbs and formulas, to identify CHM use in the NHIRD. Treatment codes were extracted from TCM clinical records of patients with IBS to assess the TCM treatment types used, including CHM and acupuncture. CHM prescription patterns were analyzed to identify the most frequently prescribed CHM for patients with IBS, the number of prescriptions, the average duration of prescriptions (in days), the average dose (in grams), and the average daily dose (in grams) was estimated. Average daily dose (g) = Average dose (g)/Average duration for prescriptions (days).

### Statistical analysis

Continuous variables were presented as means ± standard deviation, and categorical variables were presented as numbers (percentages). A multivariate logistic regression analysis was performed to examine the clinical characteristics associated with the utilization of TCM. Case was defended as TCM group and control was non-TCM group. Odd ratio (OR) was over 1 means IBS patients prefer to TCM treatment. Statistical significance was defined as a two-sided *p*-value < 0.05. The statistical analysis was conducted by SAS version 9.4 software program (SAS Institute, Inc., Cary, North. Carolina).

## Results

**FIGURE 1:**
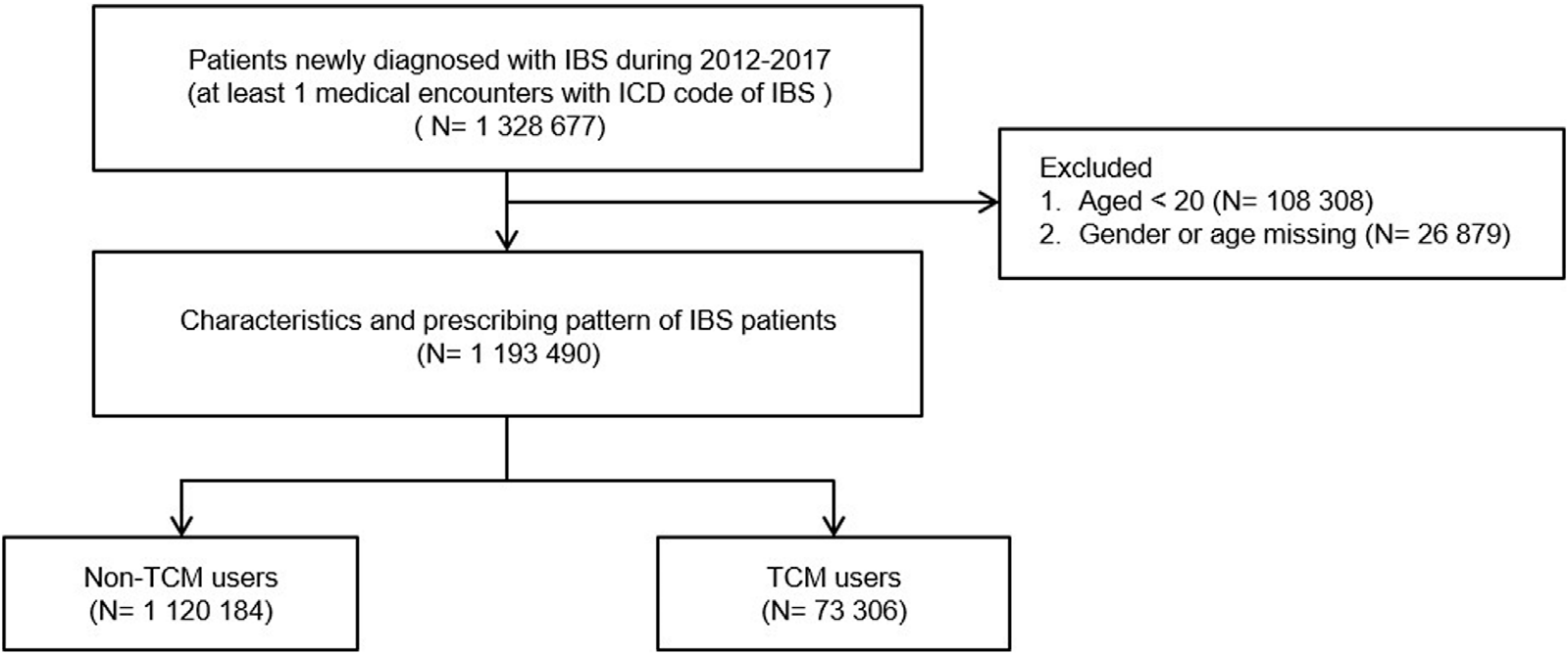
Flowchart of study population. NHIRD, National Health Insurance Research Database; ICD code, The International Classification of Diseases, Version 9/10, Clinical Modification (ICD-9-CM code: 564.1 and ICD-10-CM code: K58.0 and K58.9); IBS, Irritable bowel syndrome.

Out of the 1,193,490 patients newly diagnosed with IBS in NHIRD between 2012 and 2017, 73,306 patients (6.14%) used TCM with routine western-medicine care (TCM users) for IBS at least once during their follow-up year (Figure 1). 1,120,184 patients were non-TCM users. The female proportion was higher in both TCM and non-TCM users. Females had a significantly higher ratio of TCM use than males, with a female-to-male ratio of 1.89: 1. Additionally, the mean age of TCM users (42.94 years) was lower than that of non-TCM users (50.40 years). The age distribution of TCM users peaked in the 30–39 age group (27.29%), followed by the 40–49 (20.74%) and 20–29 age groups (20.71%), whereas the age distribution of non-TCM users peaked in the 50–59 age group (19.66%), followed by the 40–49 (17.90%) and 30–39 age groups (17.65%). Patients residing in urban areas or with an income >NT$29,000 were more likely to seek TCM consultation (Table 1).

**TABLE 1:** Demographic characteristics of IBS population with TCM care in Taiwan.

Variables	Total, n (%)	Non-TCM users, n (%)	TCM users, n (%)
Gender			
Male	546615 (45.80)	521312 (46.54)	25303 (34.52)
Female	646875 (54.20)	598872 (53.46)	48003 (65.48)
Age, mean (SD)	49.94 (17.28)	50.4 (17.32)	42.94 (15.09)
20–29	163481 (13.70)	148302 (13.24)	15179 (20.71)
30–39	217746 (18.24)	197738 (17.65)	20008 (27.29)
40–49	215684 (18.07)	200483 (17.90)	15201 (20.74)
50–59	234667 (19.66)	223197 (19.93)	11470 (15.65)
60–69	184571 (15.46)	177493 (15.84)	7078 (9.66)
≥70	177341 (14.86)	172971 (15.44)	4370 (5.96)
Urbanization (missing = 39507)			
Rural	171289 (14.35)	165149 (14.74)	6140 (8.38)
Suburban	328792 (27.55)	313346 (27.97)	15446 (21.07)
Urban	653902 (54.79)	605486 (54.05)	48416 (66.05)
Insurance premium (missing = 157734)			
>18780	227196 (19.04)	215444 (19.23)	11752 (16.03)
18780–29000	430742 (36.09)	408985 (36.51)	21757 (29.68)
29000>	377818 (31.66)	349579 (31.21)	28239 (38.52)

The comorbidities and comedication of patients with IBS receiving TCM care are shown in Table 2. The top three common GI comorbidities were gastritis and duodenitis (16.37%), peptic ulcer (15.05%), and abdominal pain (12.55%) in non-TCM users and constipation (12.17%), gastritis and duodenitis (11.68%), and peptic ulcer (10.53%) in TCM users. The three most common non-GI comorbidities were hypertension (24.84%), dyslipidemia (16.06%), and diabetes (11.8%) in non-TCM users and allergic rhinitis (12.41%), hypertension (11.74%), and dyslipidemia (9.46%) in TCM users. However, the top three psychiatric comorbidities were sleep disorder (13.68% versus 17.13%), anxiety (9.47% versus 8.17%), and depression (4.66% versus 3.41%) in both non-TCM and TCM users. Comedication patterns were similar between non-TCM and TCM users, with antacids (28.78% versus 15.29%), propulsives (24.78% versus 15%), and anxiolytics (21.19% versus 11.90%) being the top three medications.

**TABLE 2:** Baseline comorbidities and comedication of IBS population with TCM care in Taiwan.

Comorbid condition	Total, n	Non-TCM users, n (%)	TCM users, n (%)	
Gastrointestinal comorbidity				
Gastritis and duodenitis	191938	183373 (16.37)	8565 (11.68)	
Peptic ulcer	176261	168545 (15.05)	7716 (10.53)	
Abdominal pain	146880	140622 (12.55)	6258 (8.54)	
Gastroenteritis and colitis	123108	117904 (10.53)	5204 (7.10)	
Gastroesophageal reflux disease	118580	113039 (10.09)	5541 (7.56)	
Constipation	105814	96892 (8.65)	8922 (12.17)	
Gastric functional disease	87886	80595 (7.19)	7291 (9.95)	
Bloating	62410	55934 (4.99)	6476 (8.83)	
Intestinal functional disease	60234	56626 (5.06)	3608 (4.92)	
Infectious enterocolitis	36955	35278 (3.15)	1677 (2.29)	
Diarrhea	23053	21115 (1.88)	1938 (2.64)	
Inflammatory bowel disease	7257	6793 (0.61)	464 (0.63)	
Colon cancer	5992	5763 (0.51)	229 (0.31)	
Non-gastrointestinal comorbidity				
Hypertension	286886	278282 (24.84)	8604 (11.74)	
Dyslipidemia	186833	179901 (16.06)	6932 (9.46)	
Diabetes	135762	132159 (11.8)	3603 (4.92)	
Allergic rhinitis	115174	106076 (9.47)	9098 (12.41)	
Chronic lung disease	105731	101099 (9.03)	4632 (6.32)	
Fibromyalgia	97871	92531 (8.26)	5340 (7.28)	
Chronic obstructive pulmonary disease	42233	40612 (3.63)	1621 (2.21)	
Asthma	38654	37008 (3.3)	1646 (2.25)	
Chronic kidney disease	29829	29169 (2.60)	660 (0.90)	
Chronic fatigue syndrome	20791	18741 (1.67)	2050 (2.8)	
Migraine	17666	15886 (1.42)	1780 (2.43)	
Atopic dermatitis	9926	9396 (0.84)	530 (0.72)	
Obesity	3409	3163 (0.28)	246 (0.34)	
Psychiatric comorbidity				
Sleep disorder	165828	153274 (13.68)	12554 (17.13)	
Anxiety	112052	106063 (9.47)	5989 (8.17)	
Depression	54753	52256 (4.66)	2497 (3.41)	
Somatoform Disorders	17597	16911 (1.51)	686 (0.94)	
Dementia	17279	16879 (1.51)	400 (0.55)	
Psychotic disorders	11094	10621 (0.95)	473 (0.65)	
Parkinson’s disease	9820	9595 (0.86)	225 (0.31)	
Bipolar	5980	5676 (0.51)	304 (0.41)	
Stress related disorders	3023	2878 (0.26)	145 (0.20)	
Alzheimer’s disease	1530	1494 (0.13)	36 (0.05)	
Eating disorder	292	271 (0.02)	21 (0.03)	
Co-Medication				
Antacids	333592	322387 (28.78)	11205 (15.29)	
Anxiolytics	289200	277593 (24.78)	11607 (15.83)	
Propulsives	246045	237324 (21.19)	8721 (11.90)	
Laxatives	202179	195396 (17.44)	6783 (9.25)	
Simethicone	190354	183915 (16.42)	6439 (8.78)	
Proton pump inhibitors	129533	124587 (11.12)	4946 (6.75)	
Antispasmodic	129265	124926 (11.15)	4339 (5.92)	
Antidepressants	75830	72538 (6.48)	3292 (4.49)	
Antidiarrheal	49157	47492 (4.24)	1665 (2.27)	
Probiotic	52780	50849 (4.54)	1931 (2.63)	

The multiple regression analysis in Table 3 demonstrated that female (OR = 1.66; 95% confidence interval (CI), 1.63–1.69, *p* < 0.001) have a higher likelihood of utilizing TCM for IBS treatment compared to males. Otherwise, there were significantly increasing trend for visiting TCM treatments, including urban residents (OR = 1.82), insurance premiums above NT$ 29000 (OR = 1.21), peptic ulcer (OR = 1.06), gastric functional disease (OR = 1.85), intestinal functional disease (OR = 1.37), fibromyalgia (OR = 1.06), chronic obstructive pulmonary disease (OR = 1.25), chronic fatigue syndrome (OR = 1.97), migraine (OR = 1.61), obesity (OR = 1.37), sleep disorder (OR = 1.66). However, there was some factors decreasing visiting TCM treatment, the probability of seeking TCM care for IBS treatment varies among different age groups compared to the reference group (20–29 years), patients in the 30–39 age range showed a non-significant association. However, as age increases exhibited significantly decreasing for visiting TCM treatments for their IBS treatment. Moreover, also a significantly decreasing for visiting TCM treatments, including insurance premiums NT$ 18780–29000 (OR = 0.87), gastritis and duodenitis (OR = 0.84), gastroenteritis and colitis (OR = 0.74), infectious enterocolitis (OR = 0.85), hypertension (OR = 0.76), dyslipidemia (OR = 0.96), diabetes (OR = 0.75), chronic lung disease (OR = 094.), asthma (OR = 0.89), chronic kidney disease (OR = 0.79), depression (OR = 0.84), stress related disorders (OR = 0.81), and all co-medications, except for antidiarrheal drugs.

**TABLE 3:** A multiple regression analysis of factors associated with TCM care in IBS population in Taiwan.

Variables	Odds ratio (OR)	95% confidence interval (CI)	*p*-value
Gender			
Male	1		
Female	1.66	(1.63, 1.69)	<.0001
Age			
20–29	1		
30–39	0.99	(0.99, 1.04)	= 0.076
40–49	0.77	(0.96, 1.01)	<.0001
50–59	0.545	(0.75, 0.79)	<.0001
60–69	0.50	(0.53, 0.56)	<.0001
≥70	0.39	(0.48, 0.52)	<.0001
Urbanization			
Rural	1		
Suburban	1.26	(1.22, 1.30)	<.0001
Urban	1.82	(1.77, 1.87)	<.0001
Insurance Premium			
>18780	1		
18780–29000	0.87	(0.85, 0.89)	<.0001
29000>	1.21	(1.18, 1.24)	<.0001
Gastrointestinal comorbidity			
Gastritis and duodenitis	0.84	(0.82, 0.87)	<.0001
Peptic ulcer	1.06	(1.03, 1.10)	<.0001
Gastroenteritis and colitis	0.74	(0.71, 0.76)	<.0001
GERD	1.03	(1.00, 1.07)	= 0.0592
Gastric functional disease	1.85	(1.80, 1.91)	<.0001
Intestinal functional disease	1.37	(1.32, 1.43)	<.0001
Infectious enterocolitis	0.85	(0.80, 0.90)	<.0001
Non-gastrointestinal comorbidity			
Hypertension	0.76	(0.74, 0.79)	<.0001
Dyslipidemia	0.96	(0.93, 0.99)	= 0.0078
Diabetes	0.75	(0.72, 0.79)	<.0001
Chronic lung disease	0.94	(0.90, 0.97)	= 0.0002
Fibromyalgia	1.06	(1.03, 1.10)	= 0.0002
COPD	1.25	(1.18, 1.32)	<.0001
Asthma	0.89	(0.84, 0.94)	<.0001
Chronic kidney disease	0.79	(0.73, 0.87)	<.0001
Chronic fatigue syndrome	1.97	(1.87, 2.08)	<.0001
Migraine	1.61	(1.52, 1.71)	<.0001
Obesity	1.37	(1.19, 1.58)	<.0001
Psychiatric comorbidity			
Sleep disorder	1.66	(1.62, 1.70)	<.0001
Anxiety	1.02	(0.99, 1.06)	= 0.2088
Depression	0.84	(0.80, 0.88)	<.0001
Stress related disorders	0.81	(0.68, 0.98)	= 0.0318
Co-Medication			
Laxative	1.34	(0.72, 0.77)	<.0001
Antidiarrheal drugs	0.99	(0.95, 1.07)	= 0.708
Antispasmodic drugs	1.40	(0.69, 0.74)	<.0001
Probiotic	1.33	(0.64, 0.88)	= 0.0004
Antidepressant	1.18	(0.81, 0.89)	<.0001
Propulsives	1.60	(0.61, 0.65)	<.0001

Abbreviation: COPD, chronic obstructive pulmonary disease; GERD, gastroesophageal reflux disease.

The majority of TCM users (*n* = 71,999; 98.22%) received CHM, which includes both Chinese single herbs and herbal formulas (Table 4). A smaller proportion of patients (*n* = 1,082; 1.48%) received a combination of CHM and acupuncture, whereas only 92 (0.13%) patients received acupuncture alone. A total of 133 (0.18%) patients received other TCM treatments, such as moxibustion and traumatology.

**TABLE 4:** Distribution of 73306 patients with IBS with TCM care in Taiwan.

Treatments	Frequency of TCM visits n (%)			
	1–3, n (%)	4–6,n (%)	≥7, n (%)	All, n (%)
CHM	46343 (63.22)	10970 (15.24)	14686 (20.40)	71999 (98.22)
Acupuncture	82 (0.11)	4 (0.01)	6 (0.11)	92 (0.13)
CHM + Acupuncture	526 (0.72)	173 (0.24)	383 (0.52)	1082 (1.48)
Other	124 (0.17)	6 (0.01)	3 (0.00)	133 (0.18)

The top 10 Chinese single herbs were Bai-zhu (*n* = 9,123; 12.45%), Hou-pu (6,784; 9.25%), Chen-pi (6,391; 8.72%), Dan-shen (6,379; 8.7%), Yan-hu-suo (6,320; 8.62%), Fu-ling (6,098; 8.32%), Hai-piao-xiao (5,819; 7.94%), Bai-shao (5,468; 7.46%), Xiang-fu (5,280; 7.2%), and Fang-feng (5,267; 7.18%). Bai-zhu was the most frequently prescribed Chinese single herb, and Hai-piao-xiao had the longest average duration of use (90.59 days), highest average dose (114.77 g), and highest average daily dose (1.827 g) (Table 5). Jia-wei-xiao-yao-san (*n* = 16,755; 22.86%) was the most frequently prescribed Chinese herbal formula. Ban-xia-xie-xin-tang (*n* = 11,469; 14.1%) and Xiang-sha-liu-jun-zi-tang (*n* = 9,977; 12.6%) were the second and third most commonly used formulas, respectively (Table 6). Among complex formulas and sigle herbs, Jia-wei-xiao-yao-san (*n* = 16,755; 22.86%) was the most commonly used herbal medicine, and Ma-zi-ren-wan had the longest average duration of use (90.59 days) and the highest average dose (300.47 g). TCM prefers the uses of complex formuals. However, Shen-ling-bai-zhu-san had the highest average daily dose (4.817 g) (Table 6) in the treatments.

**TABLE 5:** Top 10 Chinese single herbs prescribed per person within 1 year for IBS in Taiwan (N = 73306).

Herb	Number of prescriptions, n (%)	Average duration for prescriptions (days)	Average dose (g)	Average daily dose (g)
Bai-zhu (*Atractylodes macrocephala* Koidz.)	9123 (12.45)	43.61	61.98	1.421
Hou-pu (*Magnolia officinalis* Rehd. et Wils)	6784 (9.25)	42.19	47.66	1.130
Chen-pi (*Citrus reticulata* Blanco)	6391 (8.72)	38.01	39.12	1.029
Dan-shen (*Salvia miltiorrhiza* Bge.)	6379 (8.70)	49.01	56.52	1.153
Yan-hu-suo (*Corydalis yanhusuo* W. T.Wang)	6320 (8.62)	35.20	42.18	1.198
Fu-ling (*Poria cocos* (Schw.) Wolf)	6098 (8.32)	39.17	53.76	1.372
Hai-piao-xiao (*Sepiella maindroni* de Rochebrune)	5819 (7.94)	62.82	114.77	1.827
(*Sepia esculenta* Hoyle)				
Bai-shao (*Paeonia lactiflora* Pall.)	5468 (7.46)	39.20	48.45	1.236
Xiang-fu (*Cyperus rotundus* L.)	5280 (7.20)	40.47	39.52	0.976
Fang-feng (*Saposhnikovia divaricata* (Turcz.) Schischk.)	5267 (7.18)	35.21	37.25	1.058

Average daily dose (g) = Average dose (g)/ Average duration for prescriptions (days).

**TABLE 6:** Top 10 Chinese herbal formulas prescribed per person within 1 year for IBS in Taiwan (N = 73306).

Herbal formula	Compositions	Number of prescriptions, n (%)	Average duration for prescriptions (days)	Average dose (g)	Average daily dose (g)
Jia-wei-xiao-yao-san	*Angelicae Sinensis* (Oliv.) Diels, *Atractylodes macrocephala* Koidz., *Paeonia lactiflora* Pall., *Bupleurum chinense* DC., *Poria cocos* (Schw.) Wolf, *Glycyrrhiza uralensis* Fisch., *Paeonia suffruticosa* Andr., *Gardenia jasminoides* Ellis, *Zingiber officinale* (Willd.) Rosc., *Mentha haplocalyx* Briq.	16755 (22.86)	55.39	235.08	4.244
Ban-xia-xie-xin-tang	*Pinellia ternata* (Thunb.) Breit., *Scutellaria baicalensis* Georgi, *Zingiber officinale* (Willd.) Rosc., *Panax ginseng* C. A. Mey., *Coptis chinensis* Franch., *Ziziphus jujuba* Mill., *Glycyrrhiza uralensis* Fisch.	11469 (15.65)	54.83	215.33	3.927
Xiang-sha-liu-jun-zi-tang	*Panax ginseng* C. A. Mey., *Atractylodes macrocephala* Koidz., *Poria cocos* (Schw.) Wolf, *Glycyrrhiza uralensis* Fisch., *Citrus reticulata* Blanco, *Pinellia ternata* (Thunb.) Breit., *Amomum villosum* Lour., *Aucklandia lappa* Decne., *Zingiber officinale* (Willd.) Rosc.	9977 (13.61)	63.87	264.97	4.149
Ma-zi-ren-wan	*Cannabis sativa* L., *Paeonia lactiflora* Pall., *Citrus aurantium* L., *Rheum palmatum* L., *Magnolia officinalis* Rehd. et Wils, *Prunus armeniaca* L. var. *ansu* Masim.	8985 (12.26)	90.59	300.47	3.317
Shen-ling-bai-zhu-san	*Dolichos lablab* L., *Panax ginseng* C. A. Mey., *Poria cocos* (Schw.) Wolf, *Atractylodes macrocephala* Koidz., *Glycyrrhiza uralensis* Fisch., *Dioscorea opposita* Thunb., *Nelumbo nucifera* Gaertn., *Platycodon grandiflorum* (Jacq.) A. DC., *Coix lacryma-jobi* L. var. *ma-yuen* (Roman.) Stapf, *Amomum villosum* Lour., *Ziziphus jujuba* Mill.	8559 (11.68)	45.29	218.17	4.817
Ping-wei-san	*Citrus reticulata* Blanco, *Magnolia officinalis* Rehd. et Wils, *Glycyrrhiza uralensis* Fisch., *Atractylodes lancea* (Thunb.) DC., *Zingiber officinale* (Willd.) Rosc., *Ziziphus jujuba* Mill.	7222 (9.85)	41.09	163.46	3.978
Chai-hu-shu-gan-tang	*Citrus reticulata* Blanco, *Bupleurum chinense* DC., *Paeonia lactiflora* Pall., *Citrus aurantium* L., *Glycyrrhiza uralensis* Fisch., *Ligusticum chuanxiong* Hort., *Cyperus rotundus* L.	7191 (9.81)	45.26	176.33	3.896
Qing-wei-san	*Angelica sinensis* (Oliv.) Diels, *Coptis chinensis* Franch., *Rehmannia glutinosa* Libosch., *Paeonia suffruticosa* Andr., *Cimicifuga heracleifolia* Kom.	6176 (8.42)	31.57	133.80	4.238
Wen-dan-tang	*Pinellia ternata* (Thunb.) Breit., *Bambusa tuldoides* Munro, *Citrus aurantium* L., *Citrus reticulata* Blanco, *Zingiber officinale* (Willd.) Rosc., *Glycyrrhiza uralensis* Fisch., *Poria cocos* (Schw.) Wolf, *Ziziphus jujuba* Mill.	5720 (7.80)	48.36	196.70	4.067
Li-zhong-tang	*Panax ginseng* C. A. Mey., *Glycyrrhiza uralensis* Fisch.,*Atractylodes macrocephala* Koidz., Zingiber officinale (Willd.) Rosc.	5323 (7.26)	41.87	199.55	4.766

Average daily dose (g) = Average dose (g)/Average duration for prescriptions (days).

## Discussion

This study conducted the first population-based research demonstrated the TCM prescription patterns and clinical characteristics among patients with IBS in Taiwan. 73,306 patients (6.14%) of TCM treatment with routine western-medicine care (TCM users) and 1,120,184 patients with routine western-medicine (non-TCM users) for IBS were involved. This finding is similar to that reported by Fan et al., who reported that 6.8% of patients used TCM for IBS in China (Fan et al., 2017). In Taiwan, western medicine continues to be the predominant treatment for IBS. However, TCM plays an important role in additional to western medicine, and is covered by national health insurance in Taiwan.

As a previous study in Taiwan, Chinese herbal remedies were found to be the primary choice of TCM with a utilization rate of 85.9% (Chen et al., 2007a). Similarly, CHM was the preferred treatment option for 98.22% of IBS patients. Females with IBS syndromes were more likely to seek for TCM treatment than males. This finding is consistent with previous studies (Chang & Lu, 2009; Chen et al., 2007b; C. C; Shih et al., 2012). However, the reasons behind this phenomenon were not fully explained in the earlier reports. Nevertheless, the results of previous studies proposed that independent or affluent females may have a strong belief in TCM for gynecologic problems and chronic diseases (C.-C. Shih et al., 2012). Age distribution of TCM users peaked in the 30 s. Young age has also been found to be representative of a positive attitude toward CAM in a survey of German hospitals (Huber et al., 2004). This might be because age 30 s people are mainly the breadwinners in their families, and they have more stress and possible disposable money to care for IBS since the NHI does not cover Chinese herbal pieces. Additionally, living in urbanized areas with abundant public facilities, such as easy access to public transportation, and a high density of TCM practitioners was associated with the likelihood of TCM uses (Shih et al., 2010; Yeh et al., 2016). Furthermore, choosing TCM might also be attributed to the higher expectations of patients and the fact that it allows better long-term clinical outcomes and might improve their quality of life (Figueira et al., 2010). TCM was more likely to be chosen by elderly patients for improving health conditions (Pun, 2022). For IBS patients, the morbidity for age 30–60 s are a higher plateau (>200000 patients). However, IBS patients in their 30 s had the highest visit rate to TCM clinics, and visit rates to TCM clinics for patients over 40 years old decreased obviously with age (Table 1). In sleep disorder was found to be the most prevalent, followed by anxiety and depression. Moreover, TCM can be an alternative therapy for improving sleep quality and emotional wellbeing (Aung et al., 2013). TCM practitioners typically evaluate patients’ sleep and emotions during treatment, so patients with mental health conditions are more likely to seek TCM as a healthcare option (Gureje et al., 2015).

Over 10% of IBS patients will have allergy related syndromes, such as allergic rhinitis, asthma, atopic dermatitis, etc. In diagnostic theories of TCM, the “Fei” (lungs) are believed to govern the body’s “Qi” and aid in bowel movement by regulating metabolism and waterways, Additionally, the “Fei” are closely associated with the skin. The “Da-chang” (large intestine) smoothly transport and lower waste. The “Fei” and the “Da-chang” are believed to have a close relationship in terms of their functions and interactions within the body. This connection allows for the exchange of “Qi” and other substances between the two organs, and helps to maintain balance and harmony within the body. When there is an imbalance or dysfunction in either the “Fei” or “Da-chang”, it can affect the function of the other organ as well (Wang and Zhu, 2011). While “Da-chang” is not functioning properly, such as IBS, a higher incidences of allergy related syndromes will also company (Table 2). The core patterns of these allergic syndromes can be characterized by “Fei” and “Qi” deficiencies (Tai et al., 2007; Yang et al., 2012).

Most frequently prescribed single herb for patients with IBS in Taiwan was “Bai-zhu” (Table 5), which has similar results reported as an effective agent for diarrhea-predominant IBS (IBS-D) (Park et al., 2015). Most top 10 single herbs were selected because of “Qi” circulation and reduce bloating and relieve pain caused by abdominal or muscle spasms. Research has verified the effectiveness of Tong-Xie-Yao-Fang in reducing IBS-D symptoms (M Chen et al., 2018). This formula comprises four herbals, Bai-zhu, Chen-pi, Bai-shao, and Fang-feng, which are among the top 10 Chinese single herbs prescribed for patients with IBS in Taiwan. In a previous study, it showed that Hai-piao-xiao was the most commonly prescribed single herb for patients with peptic ulcers in Taiwan (C.-Y. Huang et al., 2015). Its main component is calcium carbonate (Li et al., 2010), which may act as an antacid, helping to relieve discomfort caused by excess gastric acid. It has a significant antacid effect and is most likely to have a gratifying outcome.

The most commonly prescribed herbal formula For IBS was “Jia-wei-xiao-yao-san.” It is also commonly used to treat psychological disorders, including depression and insomnia (F. P. Chen et al., 2011; Chen et al., 2015; Park et al., 2014; Su et al., 2019). Additionally, it has been reported to be effective in regulating abnormal gastric motility and myoelectrical activity in IBS patients with functional dyspepsia (FD) (Qu et al., 2010) and ameliorating depression-like behaviors induced by chronic stress in mice by regulating the gut microbiome and brain metabolome in relation to purine metabolism (Ji et al., 2022). Brain-gut interaction is definitely affected by IBS, and numerous studies that firmly established the existence of microbiota and brain-gut interaction (Lydiard, 2001; Coss-Adame and Rao, 2014; Serafini et al., 2022). Furthermore, several studies showed a correlation among IBS with anxiety, depression, and antidepressant use (Wessely et al., 1999; Posserud et al., 2004; Lee et al., 2015). Furthermore, it may soothe the “Gan” (liver) and regulates “Qi” in TCM theories, which is beneficial for individuals aged 30–39, who are mostly working under high levels of stress. In this study, these comorbidities were common for TCM consultation, which may lead to the higher prescription rate of Jia-wei-xiao-yao-san in patients with IBS.

Ma-zi-ren-wan was reported to be effective in treating constipation and is the most commonly prescribed Chinese herbal formula for patients with this syndrome in Taiwan (Jong et al., 2010; Yang et al., 2021). The average duration of Ma-zi-ren-wan was used for the longest time and the highest dosage in average. Additionally, Ban-xia-xie-xin-tang was the second most commonly prescribed (Zeng et al., 2019). It not only relieves IBS symptoms but also provides greater benefits to FD and peptic ulcer symptoms. Consequently, it has become widely used in Taiwan for treating peptic ulcers (C. Y. Huang et al., 2015). Furthermore, Xiang-sha-liu-jun-zi-tang is frequently used for allergic related syndromes, functional abdominal pain syndrome and yields significant improvements in FD symptoms compared with prokinetic agents (Xiao et al., 2012; Liu et al., 2016). In an animal model, it promoted the expression of anti-inflammatory factors, enhanced immune response, and regulated intestinal flora, and modulated the ERK/p38 MAPK signaling pathway (Ma et al., 2019). Treatments using Shen-ling-bai-zhu-san and Chai-hu-shu-gan-tang showed significantly higher total effective rates than those of western medicine in IBS (Li et al., 2013). Moreover, an antacid effect had been reported for Shen-ling-bai-zhu-san (Wu et al., 2010). However, Ping-wei-san showed beneficial effects in reducing colonic damage in patients with colorectal cancer (Yeh et al., 2020), inhibiting inflammatory cytokine production and pathway activation of the NF-κB pathway and the NLRP3 inflammasome in mice (Zhang et al., 2019). *Magnolia officinalis* in Ping-wei-san can impede neuroinflammation and oxidative stress in the prefrontal cortex. Additionally, in a rodent model of depression, it could boost brain-derived neurotrophic factor protein levels in the hippocampus (Cheng et al., 2018; Zhang et al., 2020).

Qing-wei-san was used to treat various conditions related to heat in the stomach and blood, such as oral ulcers, periodontitis, and upper GI bleeding. It exerted anti-inflammatory effects by improving the pathological morphologies of gastric and oral mucosa in mice, reducing the levels of proinflammatory cytokines, and inhibiting the TLR4/MyD88/NF-κB signaling pathway (Shi et al., 2022). Wen-dan-tang had the potential to treat neurological and psychiatric disorders and digestive disorders (Pradhan et al., 2022). Most studies focused on insomnia and psychotic symptoms (Wu et al., 1999; Che et al., 2016; Deng and Xu, 2017; Yan et al., 2017). In Taiwan, Wen-dan-tang is commonly used for insomnia (F.-P. Chen et al., 2011). An animal study decrease insomnia-related anxiety (Wang et al., 2014). Furthermore, the inhibitory modulation of NF-κB and NLRP3 inflammasome activation by Wen-dan-tang may mediate its antidepressant effect (Jia et al., 2018). Overall, gastroesophageal reflux disease was reported to be associated with IBS (Ruigómez et al., 2009; Yarandi et al., 2010; de Bortoli et al., 2018). Wen-dan-tang consistently demonstrated significant improvement in symptom relief, and this efficacy was sustained over time in gastroesophageal reflux disease (Ling et al., 2015).

Li-zhong-tang enhanced antioxidative defense and improved mucosal immunity through the TLR-2/MyD88 signaling pathway (Song et al., 2020), it significantly restored intestinal microflora in Spleen-“Qi” deficient rats (Peng et al., 2008).

In this real-world survey, Chinese single herbs and complex formulas were suggested and may improve IBS. They are commonly prescribed to patients because they effectively alleviate comorbidities associated with IBS related disorders in physical and psychological aspects.

## Limitations

This study has some limitations. First, NHI covers Chinese herbal remedies that come in scientific granular or powder forms (extraction and preparation forms with drug certificates). Thus, the traditional form of Chinese herbal remedies (crude drugs and their complex formulas) is not covered by reimbursement and hence was excluded from this study. Second, self-pay patients were excluded from this study. Thus, the uses of TCM may be underestimated in this study. Third, as the NHIRD did not include data on the severity of IBS and specific subtypes, TCM utilization might reflect IBS clinical symptoms to a certain extent.

## Conclusion

This ethnopharmacological study reveals the prescription patterns of TCM for treating patients with IBS. The study benefits from the involvement of licensed physicians responsible for diagnosis and TCM prescriptions, increasing its credibility. Further research is needed to explore commonly used TCM formulas and single herbs. TCM can be considered for treating patients with IBS or GI disorders, as well as addressing their psychological conditions. However, clinical trials are required to support these findings.

## Data Availability

The original contributions presented in the study are included in the article/Supplementary Material, further inquiries can be directed to the corresponding authors.
