# Supplementary material for: Utilization patterns and prescription characteristics of traditional Chinese medicine among patients with irritable bowel syndrome in Taiwan
Source: Front Pharmacol. 2023 Jun 16;14:1201240. doi: 10.3389/fphar.2023.1201240 (PMC10311911; doi:10.3389/fphar.2023.1201240)
Supplement: Supplementary file 1 [file Presentation1.pdf]

Supplementary table 1. The International Classification of Diseases, Version 9/10, Clinical Modification (ICD-9-CM code and ICD-10-CM code) for comorbidities

Comorbidity	ICD-9-CM	ICD-10-CM
Gastrointestinal comorbidity		
Abdominal pain	789.0	R10. 0
Bloating	787.3	R14. 0
Colon cancer	153	C18, C7A02
Constipation	564.0	K59.00
Diarrhea	787.91	R19. 7
Gastric functional disease	5368, 5369	K30, K3189, K319
Gastritis and duodenitis	535	K29
Gastroenteritis and colitis	558	K52
Gastroesophageal reflux disease	53011, 53081	K21
Infectious enterocolitis	001–009, 11285	A00-A09, B37.82
Inflammatory bowel disease	555, 556	K50, K51
Intestinal functional disease	5648, 5649	K592, K598, K599
Peptic ulcer	531-534	K25-K28
Non-gastrointestinal comorbidity		
Allergic rhinitis	477	J30
Asthma	493	J45
Atopic dermatitis	691.8	L20. 9
Chronic fatigue syndrome	7807,	R53, G933
Chronic kidney disease	583, 585, 586, 587	N05-N07, N16, N18
Chronic obstructive pulmonary disease	491, 492, 496, 4932	J41, J42, J44
Diabetes	250	E10-E14
Dyslipidemia	272.4	E78. 5
Fibromyalgia	7291	M608, M609, M791, M797
Hypertension	401-405	I10-I15
Migraine	346	G43
Obesity	278.0, 278.01, 278.1	E65, E66
Psychiatric comorbidity		
Depression	2962–2963, 3004, 311,	F32, F33, F4321

	3090,	
Alzheimer's disease	3310	G30
Anxiety	29384, 3000, 3002, 3002, 30921	F064, F40, F41, F930
Bipolar	2964, 2965, 2966, 2967, 2968	F31
Dementia	290, 294,	F01, F02, F03
Eating disorder	3071, 30751, 30759	F50
Parkinson's disease	332	G20, G21
Psychotic disorders	295, 297, 298	F20, F22, F23, F24, F28, F29
Sleep disorder	3074, 7805	F51, G47
Somatoform Disorders	3001, 3007,3008, 306, 3078	F44, F45
Stress related disorders	308, 3098	F430, F431, F438
