# Supplementary material for: Utilization patterns and prescription characteristics of traditional Chinese medicine among patients with irritable bowel syndrome in Taiwan
Source: Front Pharmacol. 2023 Jun 16;14:1201240. doi: 10.3389/fphar.2023.1201240 (PMC10311911; doi:10.3389/fphar.2023.1201240)
Supplement: Supplementary file 2 [file Presentation2.PDF]

Supplementary table 1. Anatomical Therapeutic Chemical (ATC) code for comedications

Medication	ATC code
Antacids	A02AA02, A02AA04, A02AB, A02AB01, A02AB03, A02AB04, A02AB91, A02AB92, A02AB93, A02AB94, A02AC01, A02AD01, A02AD02, A02AD03, A02AD04, A02AF01, A02AF02, A02AG, A02AH, A02AX
Antidepressants	N06AA02, N06AA09, N06CA01, N06AB04, N06AA04, N06AA16, N06AA12, N06AB10, N06AB03, N06AB08, N06AA21, N06AB05, N06AB06
Antidiarrheal	A07XA01, A02BX05, A02BX91, A02BX12, A07XA51, A07BA01, A07BC05, A07BC30, A07XA51, A07DA03, A07BC30, A07XA04, C10AC01, C10AC02, C10AC03
Antispasmodic	A03AX08, A03BB02, A03BA01, A03CB02, A03AB91, A03AA03, A03CA02, A03AB92, A03AA07, A03AD91, A03AX05, A03AX95, A03AB02, A03BB06, A03BA03, A03CB31, A03AA04, A06AC51, A03AB12, A03BB03, A03AB06, A03AA01, A03AD01, A03AX04, A03AA30, A03AA30, A03BA92, A03AB05, A03BB01, A03AB19, A03CA01, A03AA05, A03AB20, A03AB93
Anxiolytics	N05BA01, N05BA02, N05BA03, N05BA04, N05BA05, N05BA06, N05BA08, N05BA09, N05BA11, N05BA12, N05BA16, N05BA17, N05BA22, N05BA91, N05BB01, N05BC01, N05BE01, N05BX01
Laxatives	A06AB02, A06AB20, A06AB07, A06AB05, A06AC01, A06AC51, A06AD12, A06AD11, A06AD10, A02AD01, A02AF02, A02AD04, A02AD01, A02AA04, A06AD02, A02AA02, A02AF02, A02AD01, A06AC08, A06AD15, A06AB06, A06AB20, A06AD17, A06AC03, A06AC53
Probiotic	AF07
Propulsives	A03FA01, A03FA03, A03FA04, A03FA09
Proton pump inhibitors	A02BC01, A02BC02, A02BC03, A02BC04, A02BC05, A02BC06
Simethicone	A03AX13, A02AF02
